# The role of school organizational conditions in teacher psychological resilience and stress during COVID-19 pandemic: A moderated mediation model

**DOI:** 10.3389/fpsyg.2022.1047831

**Published:** 2023-01-23

**Authors:** Chunhua Fu, Mingkun Ouyang, Xian Liu, Guilin Xu, Huimei Wang, Zhenying Ye, Jiajing Zhao

**Affiliations:** ^1^School of Education, Minzu University of China, Beijing, China; ^2^School of Education Science, Guangxi Minzu University, Nanning, China; ^3^Faculty of Health, Medicine and Life Sciences, Maastricht University, Maastricht, Netherlands; ^4^School of Marxism Studies, Wuhan Textile University, Wuhan, Hubei, China; ^5^Institute for Moral Education, Central China Normal University, Wuhan, China

**Keywords:** school organizational conditions, teacher stress, psychological resilience, perceived COVID-19 crisis strength, quantitative research

## Abstract

Educational revisions facilitate the relief of teacher stress by means of enhancing school organizational conditions. However, limited research has explored the effects of school organizational conditions on teacher stress in China. Using a sample of 734 primary and secondary school teachers from 30 provinces or municipalities of China, this study examined the effects of school organizational conditions on teacher stress in China, with a particular focus on the mediating role of psychological resilience and moderating role of perceived COVID-19 crisis strength. The results demonstrated that school organizational conditions were negatively associated with teacher stress. Furthermore, psychological resilience partially mediated the relation between school organizational conditions and teacher stress. In addition, perceived COVID-19 crisis strength significantly moderated the direct and indirect relations between school organizational conditions and teacher stress. The relations between school organizational conditions and teacher stress and between school organizational conditions and psychological resilience were stronger for teachers who perceived low levels of COVID-19 crisis strength. However, the indirect relation between psychological resilience and stress was stronger for teachers who perceived high levels of COVID-19 crisis strength. Implications have been provided accordingly.

## 1. Introduction

School operations have been severely interrupted since the outbreak of COVID-19, resulting in school closure in as many as 191 countries around the world and affecting 91.3% of the school population (The United Nations Educational, Scientific and Cultural Organization [UNESCO], 2020). The pandemic has been a massive and unprecedented disruption for most teachers’ regular lives (Espino-Díaz et al., 2020). Ozamiz-Etxebarria et al. (2021) have found a high proportion of teachers suffering from stress caused by COVID-19. Teacher stress, an unpleasant emotional experience with psychological syndromes from work, including anger, frustration, anxiety, tension and depression (Kyriacou, 1987), has been drawing attentions of scholars around the globe (Stoeber and Rennert, 2008; Skaalvik and Skaalvik, 2015; Newberry and Allsop, 2017). Many studies have proclaimed being a teacher as one of the most stressful jobs (Bauer et al., 2007; Chaplain, 2008; Liu and Onwuegbuzie, 2012). However, teachers may endure various long-term problems under high stress from work, causing issues like the outflow of teachers (Clark and Antonelli, 2009; Skaalvik and Skaalvik, 2011; Struyven and Vanthournout, 2014) and damage to students’ academic achievements and mental health (Shen et al., 2015; Herman et al., 2018; Sutcher et al., 2019).

Teachers’ working conditions in China have been deteriorating since the COVID-19 pandemic. To mitigate teachers’ stress in such context, delineating pivotal predictors is crucial for releasing teachers’ stress. Given the importance of school organizational conditions to teachers’ mental health, this study targeted to examine the association between school organizational conditions and teacher stress. Further, this study aimed at exploring teacher psychological resilience as one possible mechanism through which school organizational condition might relate to teacher stress. Additionally, as individuals often have different experiences with COVID-19, we also intended to explore how individuals’ perceptions of COVID-19 might moderate the above relationship among organizational conditions, psychological resilience, and teacher stress. The current study was situated in the Chinese context, where primary and secondary school teachers tend to suffer from high working pressure due to large class sizes, heavy workload, and heavy emphasis on academic performance by parents and society as a whole (Liu and Onwuegbuzie, 2012; Wang et al., 2015) during the pandemic. This study will extend our understanding of the association between school organizational conditions and teacher stress in non-Western cultures.

## 2. Literature review

### 2.1. Organizational conditions and teacher stress

School organizational situations have been identified as an important influencing factor of teacher stress (Parent-Lamarche and Marchand, 2019) and are generally conceptualized as a great number of organizational factors in school denoting the organization of educational processes and people (Creemers and Reezigt, 1996).

Teacher stress originates from mainly two aspects in existing literature: inadequate work-related demands and school-based support. Work-related stressors include and are not limited to student behavior management, tremendous workload, inter-relationship with colleagues and students, and so on (Tsivgiouras et al., 2019). A high volume of work and lack of time often occurs when teachers’ abilities cannot meet the requirements of daily teaching (Torres et al., 2009). Meanwhile, Ouellette et al. (2018) found that support from colleagues, especially from school leaders, could alleviate teacher stress. The support from colleagues and their network are natural psychological resources for educators (Kaihoi et al., 2022).

Although many studies have explored the role of school organizational conditions in teacher stress, existing studies often only focus on one aspect of school organizational conditions. For instance, leadership, working atmosphere, and organizational structure might be potential dimensions to evaluate organizational conditions in school (Zhang and Zheng, 2020; Nong et al., 2022). The existing literature about Chinese teachers had explored the association between empowering leadership and stress (Nong et al., 2022) and relationship between organizational support and burnout (Xu and Yang, 2018). However, empirical understanding of the independent impact of different aspects of school organizational conditions on teacher stress is limited. Therefore, more research should be conducted to verify the holistic role of school organizational conditions on teacher stress in China.

### 2.2. Psychological resilience

Extending upon research on the association between school organizational conditions and teacher stress, some researchers have explored possible mechanisms behind such association. Durbin et al. (2019) proposed that psychological resilience could be an important mechanism to consider, in virtue of the nature of psychological resilience, as a dynamic process of adaptation and swift recovery against adversity and trauma owing to capacities for navigation to social, cultural and psychological contexts (Ungar, 2011; Windle, 2011; Stainton et al., 2018). Positive adaptation and adversity were two key components that were frequently mentioned among all definitions by academics (Fletcher and Sarkar, 2013). Teachers’ interpersonal communication within the context of schools can be demonstrated as a positive process of adaptation (Howe et al., 2012), and stress from occupation and COVID-19 exists as extensive adversity among primary and secondary teachers. High resilience can be regarded as an essential personal asset that revives teachers from adversity and relieves teachers from mental stress (Deng et al., 2020). This provides support for our exploration of the mediating role of psychological resilience in the association between school organizational conditions and teacher stress.

Teaching is an occupation with high stress because of its requirement for emotional interactions (Johnson et al., 2005). However, personal and psychological behaviors are not the only determinants of personal requirements or aims, social and organizational factors also play an important role (Salancik and Pfeffer, 1978). Besides support from families and friends as the primary resource, colleagues and supervisors can be the most productive resource that helps reduce teachers’ stress (Zhang and Zhu, 2007). A systematic review by Kangas-Dick and O’Shaughnessy (2020) suggested that school-level contextual factors appear to be the most essential to contribute to psychological resilience outweighing personal factors for the thrive of teachers’ well-being. Hu et al. (2019) also demonstrated that school conditions and relational trust were significant predictors of teacher resilience, which has not been paid much attention to in previous studies.

Therefore, increasing psychological resilience can be an indirect effect between support teachers receive from school leaders and colleagues and enhancing teachers’ mental health (Guo et al., 2020). However, there was limited research regarding the mechanism and process and it is what our study aimed at.

### 2.3. Perception of teacher stress during the pandemic

Considerable studies have explored teachers’ feelings and stress during COVID-19 (Robinson et al., 2022). Against the background of COVID-19 pandemic, extra efforts are needed for daily teaching activities (Nagasawa and Tarrant, 2020). As a disruptive event, the pandemic brings an abundance of the likelihood of negative mental and physical health (Subica et al., 2012), as many studies have examined COVID-19 as a determinant on teacher stress (e.g., Baker et al., 2021; Diliberti et al., 2021; Steiner and Woo, 2021).

Nevertheless, significant individual differences in the experience and perception of COVID-19 may directly associate individual well-being or interact with other factors to jointly associate with individual well-being. From the perspective of event system theory, the strength of a salient event to individuals can be reflected in its novelty, disruption, and criticality (Morgeson et al., 2015). In the context of the COVID-19 pandemic, the novelty can be considered as an unexpected impact on teachers; disruption is the extent to which teachers are intruded and hindered by the virus; and criticality reflects the correlation between COVID-19 and individual career development in the long term (Morgeson et al., 2015). These perspectives vary enormously owing to personal traits and external assets and so on and associate with individual mental stress.

Previous studies have investigated teachers’ perception toward school community needs (Hatzichristou et al., 2021), classroom safety (Quansah et al., 2022) and online learning (Konukman et al., 2022) during COVID-19. However, few studies analyzed the perceived strength of COVID-19 as a disruptive crisis and its association with teachers’ working conditions and mental health in China. Given previous literature, there is a possibility that perceived COVID-19 crisis strength may be a moderator in the model that the current study developed.

### 2.4. The present study and hypotheses

We explored how teacher stress is associated with different school organizational factors, including the mediated contributions of psychological resilience to educator stress. Based on the empirical research described, the following hypotheses were formulated.

*H1*: School organizational conditions negatively predict teacher stress.

*H2*: School organizational conditions are indirectly associated with teacher stress by means of psychological resilience.

**Figure 1:**
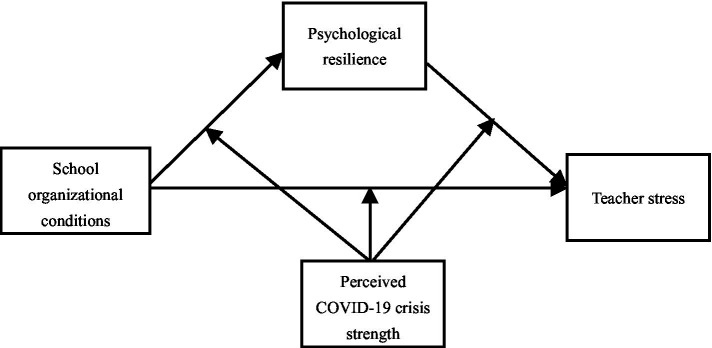
The assumptive moderated mediation model.

*H3*: Teachers’ perceived COVID-19 crisis moderates the processes between school organizational conditions, psychological resilience and teacher stress. Figure 1 shows the assumptive moderated mediation model of this study.

## 3. Materials and methods

### 3.1. Participants

This study involved samples of 734 teachers from 30 provinces or municipalities in China. Teachers completed an online survey between June 2022 to August 2022. The teacher participants illustrated variances in gender, age, teaching tenure, years of schooling and teaching institutions (see Table 1). Convenience sampling method was adopted in this study. Participants are teachers working in primary and secondary schools in China. We contacted the principals in the local educational department from different provinces and districts in China, such as education bureaus and schools, to increase the diversity of participants. Participants are teachers working in primary and secondary schools in China. There are no additional exclusion criteria on age, gender, race or teaching subjects. If the participants did not complete the questionnaire, or filled in the questionnaire indiscriminately, these data have been removed from the samples.

**Table 1:** Demographic information of the teachers.

Variables	Levels	*n*	%	Cumulative %
Gender	Male	143	19.50%	
	Female	591	80.50%	100
Age	Less than 35 years	423	57.60%	
	35–50 years	205	27.90%	85.5
	More than 50 years	106	14.50%	100
Teaching experience	Less than 13 years	450	61.30%	
	13–26 years	139	18.90%	80.2
	More than 26 years	145	19.80%	100
Teaching school	Primary school	426	58.00%	
	Junior high school	140	19.10%	77.1
	Senior high school	168	22.90%	100

### 3.2. Measures

#### 3.2.1. School organization conditions

School organization conditions were measured by the subscale of the School Organization Conditions Scale (SOCS) developed by Zhang and Sun (2018). The subscale includes 16 items and comprises three dimensions: 6 items about supportive leadership (e.g., “School leaders provide us information and resources related to teaching.”), 5 items about collaborative atmosphere (e.g., “Teachers are willing to exchange and collaborate with each other in our school.”), and 5 items about organizational structure (e.g., “School leaders participate in our collaborative learning activities.”). All the items were rated on a 6-point Likert scale ranging from 1 (*strongly disagree*) to 6 (*strongly agree*). Higher average scores indicate more positive school organization conditions perceived by teachers. The subscale of the SOCS had good construct validity, as the first-order CFA model generated a good fit (*χ*^2^/*df* = 4.49, *p* < 0.001; RMSEA = 0.069; CFI = 0.96, TLI = 0.95). In this study, Cronbach’s alpha for the subscale was 0.91.

#### 3.2.2. Teacher stress

Teacher Stress Scale (TSS) was used to assess teachers’ stress (Luo et al., 1997; Chen et al., 2022). The TSS consists of three dimensions, namely inadequate school-based support (3 items, e.g., “I felt stressed for not having support from colleagues at my school.”), teaching-related demands (4 items, e.g., “I felt stressed for not being able to meet the diverse learning needs of my students.”), and interpersonal stress (4 items, e.g., “Most colleagues are not interested or friendly to me.”). All the items were rated by teachers on a 5-point Likert scale ranging from 1 (*strongly disagree*) to 6 (*strongly agree*), with higher average scores indicating higher levels of teacher stress. First-order CFA results validated the construct of the TSS with a good model fit (*χ*^2^/*df* = 9.98, *p* < 0.01; RMSEA =0.07; CFI =0.96, TLI =0.93). In this study, Cronbach’s alpha for the scale was 0.94.

#### 3.2.3. Psychological resilience

Brief Resilience Scale (BRS) was used to measure teachers’ psychological resilience (Smith et al., 2008). The BRS includes six items (e.g., “It does not take me long to recover from a stressful event.”). All the items were rated by teachers on a 5-point Likert scale ranging from 1 (*strongly disagree*) to 5 (*strongly agree*). Higher average scores indicate higher levels of psychological resilience. First-order CFA results showed that BRS had good construct validity (*χ*^2^/*df* = 1.41, *p* > 0.05; RMSEA = 0.02; CFI = 0.99, TLI = 0.99). In this study, Cronbach’s alpha for the scale was 0.62.

#### 3.2.4. Perceived COVID-19 crisis strength

The Perceived COVID-19 Crisis Strength Scale (PCCS) developed by Liu et al. (2021) was used to assess teachers’ perceived COVID-19 crisis strength. The PCCS includes 11 items and three dimensions, namely, novelty (4 items, e.g., “There is an understandable sequence of steps that can be followed in responding to this COVID-19 crisis.”), disruption (4 items, e.g., “This COVID-19 crisis causes me to stop and think about how to respond.”), and criticality (3 items, e.g., “This COVID-19 crisis is of a priority to me.”). All the items were rated on a 5-point Likert scale ranging from 1 (*strongly disagree*) to 5 (*strongly agree*). Mean scores across the 11 items were used in the present, with higher scores meaning higher levels of perceived COVID-19 crisis strength. In the perceived COVID-19 crisis strength scale, the first-order CFA model generated a good fit (*χ*^2^/*df* = 4.134, *p* < 0.001; RMSEA = 0.065; CFI = 0.998, TLI = 0.97), indicating that this scale had acceptable construct validity. In this study, Cronbach’s alpha for the scale was 0.89.

### 3.3. Data analysis

Second, this study used Model 4 of the PROCESS macro developed by Hayes (2013) to examine the mediating effect of psychological resilience in the relationship between school organization conditions and teacher stress (see Table 2). Third, this study further examined whether the mediation process was moderated by perceived COVID-19 crisis strength using Hayes’s (2013) PROCESS macro (Model 59; see Table 3). The bootsrapping method based on 5,000 resample was used to examine the significance of the direct and indirect effects.

**Table 2:** The mediation effect of school organizational conditions on teacher stress *via* resilience.

Variables	Model l (Teacher stress)				Model 2 (Psychological resilience)				Model 3 (Teacher stress)			
	*β*	SE	LLCI	ULCI	*β*	SE	LLCI	ULCI	*β*	SE	LLCI	ULCI
Gender	−0.002	0.09	−0.010	0.005	0.053	0.091	−0.125	0.231	0.024	0.081	−0.134	0.183
Age	0.009	0.01	−0.008	0.022	0.004	0.011	−0.018	0.027	0.012	0.01	−0.009	0.032
Teaching tenure	−0.017	0.01	−0.010	0.008	−0.002	0.01	−0.022	0.018	−0.018^*^	0.009	−0.035	−0.0005
School organizational conditions	−0.228^***^	0.04	−0.139	−0.008	0.314^***^	0.036	0.244	0.384	−0.074^*^	0.033	−0.139	−0.008
Psychological resilience									−0.496^***^	0.033	−0.561	−0.431
*R^2^*	0.063				0.100				0.284			
*F*	9.732^***^				16.100^***^				48.080^***^			

*β* are standardized coefficients. SE, standard error; LLCI, lower limit of the 95% confidence interval; ULCI, upper limit of the 95% confidence interval. Each column is a regression model that predicts the criterion at the top of the column. ^*^p < 0.05, ^***^p < 0.001.

**Table 3:** The moderated mediation effect of school organizational conditions on teacher stress.

Variables	Model 1 (Psychological resilience)				Model 2 (Teacher stress)			
	*β*	SE	LLCI	ULCI	*β*	SE	LLCI	ULCI
Gender	0.025	0.084	−0.141	0.19	0.065	0.076	−0.085	0.215
Age	0.007	0.011	−0.014	0.027	0.01	0.01	−0.009	0.029
Teaching tenure	−0.006	0.009	−0.024	0.012	−0.015	0.008	−0.031	0.001
School organizational conditions	0.264^***^	0.033	0.198	0.329	−0.076^*^	0.032	−0.137	−0.014
Perceived COVID-19 crisis strength	−0.324^***^	0.034	−0.391	−0.257	0.300^**^	0.033	0.235	0.366
School organizational conditions * Perceived COVID-19 crisis strength	−0.093^**^	0.033	−0.159	−0.03	0.100^**^	0.032	0.038	0.162
Psychological resilience					−0.385^***^	0.033	−0.451	−0.319
Resilience * Perceived COVID-19 crisis strength					−0.074^**^	0.027	−0.126	−0.021
*R^2^*	0.224				0.371			
*F*	35.020^***^				53.451^***^			

*β* are standardized coefficients. SE, standard error; LLCI, lower limit of the 95% confidence interval; ULCI, upper limit of the 95% confidence interval. Each column is a regression model that predicts the criterion at the top of the column. ^*^p < 0.05, ^**^p < 0.01, ^***^p < 0.001.

## 4. Results

### 4.1. Common method bias

As all measures were completed by teachers, we examined the severity of the common method bias issue by using Harman’s single factor test. The total variance for a single factor was 32.761% after all items were loaded into one common factor, which was less than the 50% cutoff suggested by Mat Roni (2014). It indicated that a common method bias was not influential to the data, so as to the results. So, the common method biases are not required in the current study.

### 4.2. Descriptive statistics and correlation analysis

As presented in Table 4, school organizational conditions were negatively correlated with teacher stress (*r* = −0.228, *p* < 0.001) and perceived COVID-19 crisis strength (*r* = −0.143, *p* < 0.001), but positively correlated with psychological resilience (*r* = 0.310, *p* < 0.001). Teacher stress was negatively correlated with psychological resilience (*r* = −0.521, *p* < 0.001) and positively correlated with perceived COVID-19 crisis strength (*r* = 0.456, *p* < 0.001). In addition, psychological resilience was negatively correlated with perceived COVID-19 crisis strength (*r* = −0.382, *p* < 0.001).

**Table 4:** Descriptive statistics of and correlations among study variables.

	*M*	SD	1	2	3	4
1. School organizational conditions	4.109	0.761	1			
2. Teacher stress	2.843	0.953	−0.228^***^	1		
3. Psychological resilience	3.414	0.608	0.310^***^	−0.521^***^	1	
4. Perceived COVID-19 crisis strength	2.865	0.553	−0.143^***^	0.456^***^	−0.382^***^	1

^***^p < 0.001.

### 4.3. Mediation effect of psychological resilience

Model 4 of the PROCESS macro (Hayes, 2013) was employed to examine the mediating effect of psychological resilience on the relationship between school organizational conditions and teacher stress. The results of the mediation analysis were displayed in Table 2, showing that school organizational conditions was negatively correlated with teacher stress (*β* = −0.228, *p* < 0.001) after controlling for covariates of gender, age, and teaching tenure (see Model 1). Results also presented that school organizational conditions was positively correlated with psychological resilience (*β* = 0.314, *p* < 0.001; see Model 2), which was negatively correlated with teacher stress (*β* = −0.496, *p* < 0.001; see Model 3). The indirect effect of school organizational conditions on teacher stress by means of psychological resilience was significant (ab = −0.156, SE = 0.024, 95%CI = [−0.208, −0.110]), and the direct effect was also significant (*c*’ = −0.074, SE = 0.033, 95%CI = [−0.139, −0.007]). Thus, the partially mediating effect of psychological resilience was supported, which accounted for 67.83% of the total effect.

### 4.4. Moderation effect of perceived COVID-19 crisis strength

**Figure 2:**
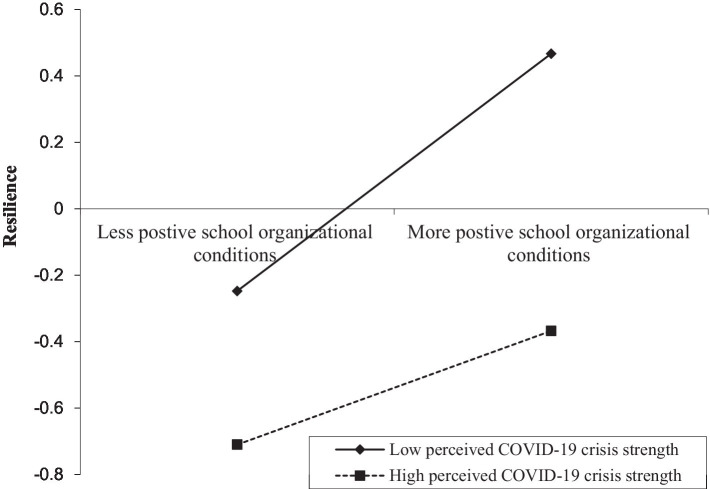
Perceived COVID-19 crisis strength moderates the indirect relationship between school organizational conditions and psychological resilience.

We used Model 59 of the PROCESS macro (Hayes, 2013) to test the moderating effect of perceived COVID-19 crisis strength on the direct and indirect relationships between school organizational conditions and teacher stress. The results of the moderated mediation were presented in Table 3. After controlling for covariates, Model 1 indicated that school organizational conditions had a positive association with psychological resilience (*β* = −0.324, *p* < 0.001), and this effect was significantly moderated by perceived COVID-19 crisis strength (*β* = −0.093, *p* < 0.01). Thus, the relationship between school organizational conditions and psychological resilience was moderated by perceived COVID-19 crisis strength. To better understand this moderation effect, we plotted predicted psychological resilience against school organizational conditions, for low and high levels of perceived COVID-19 crisis strength (1 SD below the mean and 1 SD above the mean, respectively; see Figure 2). Simple slope tests demonstrated that for teachers with perceived low levels of COVID-19 crisis strength, the positive relationship between school organizational conditions and psychological resilience was stronger (*β*_simple_ = 0.357, *p* < 0.001). However, for teacher with perceived high levels of COVID-19 crisis strength, the positive relationship between school organizational conditions and psychological resilience was weaker (*β*_simple_ = 0.171, *p* < 0.001).

**Figure 3:**
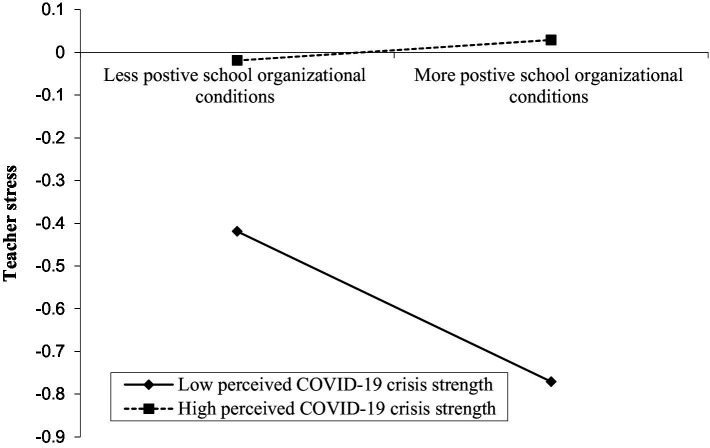
Perceived COVID-19 crisis strength moderates the direct relationship between school organizational conditions and teacher stress.

In addition, the results of Model 2 displayed that school organizational conditions was negatively correlated with teacher stress (*β* = −0.076, *p* < 0.05), and this relationship was significantly moderated by perceived COVID-19 crisis strength (*β = 0*.100, *p* < 0.01). As shown in Figure 3, we plotted predicted teacher stress against school organizational conditions, for low and high levels of perceived COVID-19 crisis strength (1 SD below the mean and 1 SD above the mean, respectively). Simple slope tests showed that for teachers with perceived low levels of COVID-19 crisis strength, school organizational conditions was negatively correlated with teacher stress (*β*_simple_ = −0.176, *p* < 0.001); while for teachers with perceived high levels of COVID-19 crisis strength, the relationship between school organizational conditions and teacher stress was not significant (*β*_simple_ = −0.024, *p* = 0.581).

**Figure 4:**
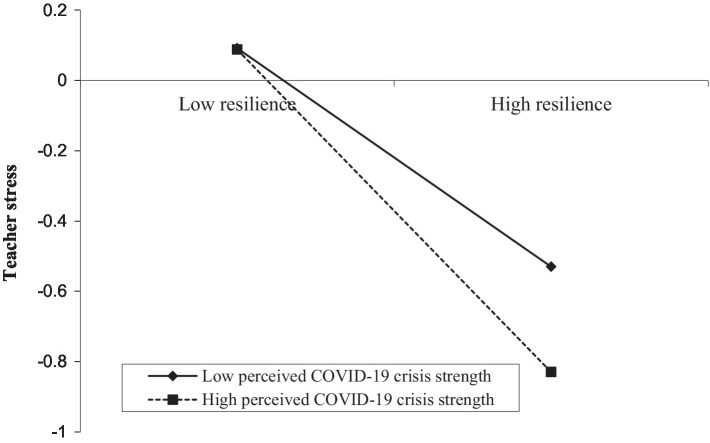
Perceived COVID-19 crisis strength moderates the indirect relationship between psychological resilience and teacher stress.

Besides, the results of Model 2 also showed that psychological resilience was negatively correlated with teacher stress (*β =* −0.385, *p* < 0.001), and this relationship was significantly moderated by perceived COVID-19 crisis strength (*β = −0*.074, *p* < 0.01). We plotted predicted teacher stress against psychological resilience, for low and high levels of perceived COVID-19 crisis strength (1 SD below the mean and 1 SD above the mean, respectively; see Figure 4). Simple slope tests showed that for teachers with perceived low levels of COVID-19 crisis strength, the negative relationship between psychological resilience and teacher stress was weaker (*β*_simple_ = −0.311, *p* < 0.001). However, for teachers with perceived high levels of COVID-19 crisis strength, the negative relationship between psychological resilience and teacher stress was stronger (*β*_simple_ = −0.459, *p* < 0.001).

The bias-corrected percentile bootstrap analyzes further confirmed that the indirect effect of school organizational conditions on teacher stress by means of psychological resilience was moderated by perceived COVID-19 crisis strength. Specifically, for teachers with perceived low levels of COVID-19 crisis strength, the indirect relationship between school organizational conditions and teacher stress was stronger (*β* = −0.111, SE = 0.023, 95%CI = [−0.162, −0.069]). While for perceived high levels of levels of COVID-19 crisis strength, the indirect relationship between school organizational conditions and teacher stress was weaker (*β* = −0.078, SE = 0.029, 95%CI = [−0.146, −0.029]).

## 5. Discussion

In the present study, we explored the association between school organizational conditions and teacher stress, with a mediated role of psychological resilience and moderated role of perceived COVID-19 crisis stress. We obtained significant support from the analysis above concerning research hypotheses.

### 5.1. Effects of school organizational conditions on teacher stress

Our findings provided insights into the relationship between school organizational conditions and teacher stress in the Chinese context. This study showed that Chinese primary and secondary teachers would be more relieved from working stress when their schools provided more support on organizational conditions. In line with similar studies focused on school teachers in Western societies, organizational and environmental factors significantly correlated with teacher stress (Clayback and Williford, 2021; Nikolopoulou and Kousloglou, 2022). The research findings were also in accordance with previous studies indicating that positive cultural, social and collaborative support from school can reduce the possibility of teacher stress (Kyriacou, 2001; Paterson and Grantham, 2016). As indicated in previous literature, beneficial school organizational conditions could promote teachers’ well-being and personal development in the context of primary and secondary schools (Frieder et al., 2018); on the contrary, the lack of system support and insufficient school organizations could be correlated with teacher stress (Tebben et al., 2021).

The higher level of school organizational conditions increased teachers’ chance to interact and socialize with colleagues and principles at school, which may create a positive cycle between individual health and school organizational conditions in China. As the rights and duties of Chinese teachers had been determined from the perspectives of authority, hierarchy and responsibility, these are not the same as those in informal groups (Çimili Gök, 2022). The Chinese tradition of collectivism caused the high power distance nature of school top leaders (Iwasaki, 2005). The principals and leaders in the school organization have been empowered the ability to contribute assets and resources for teachers. Teachers needed to obey the administration from the upper level, otherwise, educational resources may not be provided sufficiently without a good relationship, which may lead to teacher stress (Schoen, 2005). Therefore in Chinese primary and secondary schools, school-level contextual factors outweighed individual factors contributing to teachers’ mental health in China (Kangas-Dick and O’Shaughnessy, 2020).

### 5.2. Mediating role of psychological resilience

This study proved the mediating role of psychological resilience concerning the relationships between school organizational conditions and teacher stress. First, in line with previous studies (Salancik and Pfeffer, 1978; Stainton et al., 2018), this study confirmed that psychological resilience as a critical psychological condition showed a positive correlation with school organizational conditions in China. Owing to the stress from school regulations, governmental policies, social environment and other aspects, Chinese teachers were usually required to focus on enhancing professional skills and cultivating students’ core competencies as much as possible to help students master knowledge, which lead to ignorance of teachers’ ability of coping with adversities on constructing psychological and mental health to some extent (Wang et al., 2022). The competitive circumstance not only existed in enhancing students’ academic performance, but also transferred into Chinese teachers’ relationships owing to evaluation of professional titles and salary, impeding collaborative support from colleagues (Zhang and Li, 2021). In addition, the lack of mental support from school weakened teachers’ ability to cope with afflictions and adversities. Heavy workload expended teachers’ physical and psychological energy, required long working time at schools and limited the possibility of acquiring emotional support from families and friends, which negatively predicted psychological resilience of Chinese teachers (Guo et al., 2020).

Second, this study revealed that psychological resilience can negatively predict teacher stress. As indicated in previous literature, psychological resilience was internal capabilities enabling individuals’ capability to cope with people and the surrounding environment (Stainton et al., 2018). Teachers with the belief of potential benefits from stress, namely psychological resilience toward teacher stress, experienced a lower level of stress and teacher attrition (Kim and Asbury, 2020). Accordingly, this study added credence to Deng et al.’s (2020) opinions that psychological resilience was a personal ability and asset to revive teachers from adversity, burnout and mental stress at work. From the perspective of role socialization theory, psychological resilience enhanced interpersonal connections and increases teachers’ competence to cope with people’s reactions and exceptions. Therefore, the result proved validity to the findings of Guo et al. (2020) that psychological resilience can be a significant mediating effect between teacher supports from school leaders and colleagues and teacher stress in the Chinese context. Compared with a single impact of external factors or internal factors on teacher stress, this study attempted to integrate organizational and individual impact as an indirect correlation to expand the source of teacher stress in this field of existing literature.

### 5.3. Moderating role of perceived COVID-19 crisis strength

This study provided insights into the moderating effects of teachers’ perceived COVID-19 crisis strength on the associations among school organizational conditions, psychological resilience and teacher stress. First, high-level perceived COVID-19 crisis strength weakened the positive correlation between school organizational conditions and psychological resilience. Owing to extra efforts needed to be made to deal with administrative issues during the pandemic, school organizational conditions were reshaped by the pandemic and the administration was not able to provide teachers with psychological support contributing to teacher resilience from limited resources (Kangas-Dick and O’ Shaughnessy, 2020); we suspected that with higher perceived COVID-19 crisis strength, teachers’ mental assets were occupied by coping with the pandemic individually, while losing confidence on the support from schools (Diliberti et al., 2021). The negative perception might prevent teachers from benefiting psychologically from supportive organizational conditions.

Second, high-level perceived COVID-19 crisis strength weakened the correlation between school organizational conditions and teacher stress. With higher perceived COVID-19 crisis strength, nonsignificant correlations had been testified according to the results from data collected among Chinese primary and secondary teachers. When teachers’ perceived strength of COVID-19 crisis are relatively high, it might obstruct teachers communicating with colleagues and leaders, the lack of occupational support might aggravate teachers’ workload and individual stress (Cross et al., 2004). In other words, school organizational conditions could not negatively predict teacher stress if and when teachers perceived COVID-19 as an important event owing to higher perception of COVID-19 impeding the organizational support from school, which is in accordance with the discussion above (Cann et al., 2022).

Third, high-level perceived COVID-19 crisis strength strengthened the negative correlation between psychological resilience and teacher stress. When teachers perceived COVID-19 as a stronger crisis, the negative association between individual psychological resilience and teacher stress was stronger, which means psychological resilience works more efficiently as a protective factor against teacher stress when teachers reported higher perceived COVID-19 crisis strength. Community resilience (Saghin et al., 2022) and nurses’ resilience (Balay-Odao et al., 2021) have been enhanced facing the crash of COVID-19, and people even made fun of the virus and appreciating medical staff’s dedication on social networks (Sadeghmoghadam et al., 2020). This may be explained by a psychological dependence against a public crisis to a group because people obtained a sense of belonging which enhanced teachers’ psychological health and social cohesion.

## 6. Limitations and directions for future research

This study has several limitations that warrant attention. First, a convenience sample was used, and therefore, the current findings cannot be generalized nationwide. Therefore, domestic and foreign representative samples should be used in future studies to generalize to other cultures. Second, the data collected in this study are cross-sectional and cannot prove causality between school organizational conditions, psychological resilience, perceived COVID-19 crisis strength and teacher stress. Prospective studies are suggested to employ longitudinal research designs to explore causalities long-term associations between factors. Third, since teacher stress was self-reported, which may cause uncontrolled effects of common method bias. Other methods should be used to examine the effects between variables more accurately in future research. Objective evaluations of school organization conditions should be used to better delineate their effects on teacher psychological resilience and teacher stress. Fourth, the current study focused on psychological resilience as a mechanism. Other possible mechanisms may include job motivation and self-esteem, which need to be examined in future research.

## 7. Conclusion and implications

This study provides some practical implications for improving the organizational conditions and relieving teacher stress in Chinese primary and secondary schools. First, school leaders should focus their leadership practice on improving school organizational conditions, such as providing professional guidance on daily classes, bolstering teachers’ opportunities for communication and mutual trust and organizing meeting of collaborative learning and educational resources. Second, school psychologists and administrators should provide incremental behavioral and social support to obtain nurture of teacher resilience, assisting teachers to cope with mental problems and relieve stress within school organizational structure (Oducado et al., 2021). Third, attentions should be paid to lessening perceived COVID-19 crisis strength by strengthening the psychological construction of teachers, specifically proceeding from changing teachers’ attitude toward COVID-19, which may help individuals put psychological assets into how to make better use of resources around them (such as good school support environment) to improve their own mental health.

From the theoretical perspective, the present findings provide an empirical framework to test the mediating role of psychological resilience in the association between school organizational conditions and teacher stress and the moderating role of perceived COVID-19 crisis strength in the links among these factors. This framework sheds light on the mechanism underlying the relationship between school organizational conditions and teacher stress.

## Data availability statement

The raw data supporting the conclusions of this article will be made available by the authors, without undue reservation.

## Ethics statement

The studies involving human participants were reviewed and approved by Research Ethics Committee of Minzu University of China. The patients/participants provided their written informed consent to participate in this study.

## Author contributions

CF, MO, and XL: conceptualization, software, and writing original draft. GX and XL: writing, supervision, and validation. HW, ZY and JZ: review and editing. All authors contributed to the article and approved the submitted version.

## Conflict of interest

The authors declare that the research was conducted in the absence of any commercial or financial relationships that could be construed as a potential conflict of interest.

## Publisher’s note

All claims expressed in this article are solely those of the authors and do not necessarily represent those of their affiliated organizations, or those of the publisher, the editors and the reviewers. Any product that may be evaluated in this article, or claim that may be made by its manufacturer, is not guaranteed or endorsed by the publisher.
